# Genetic Responses and Aflatoxin Inhibition during Co-Culture of Aflatoxigenic and Non-Aflatoxigenic *Aspergillus flavus*

**DOI:** 10.3390/toxins13110794

**Published:** 2021-11-11

**Authors:** Rebecca R. Sweany, Brian M. Mack, Geromy G. Moore, Matthew K. Gilbert, Jeffrey W. Cary, Matthew D. Lebar, Kanniah Rajasekaran, Kenneth E. Damann Jr.

**Affiliations:** 1 Food and Feed Safety Research Unit, Southern Regional Research Center, US Department of Agriculture, New Orleans, LA 70124, USA; brian.mack@usda.gov (B.M.M.); matthew.gilbert@usda.gov (M.K.G.); jeff.cary@usda.gov (J.W.C.); matthew.lebar@usda.gov (M.D.L.); 2 Department of Plant Pathology and Crop Physiology, Louisiana State University, Baton Rouge, LA 70808, USA; kdamann@agcenter.lsu.edu

**Keywords:** aflatoxin, secondary metabolism, fungal interactions, biocontrol, biocontrol mechanism, RNA-seq, toxin inhibition

## Abstract

Aflatoxin is a carcinogenic mycotoxin produced by *Aspergillus flavus*. Non-aflatoxigenic (Non-tox) *A. flavus* isolates are deployed in corn fields as biocontrol because they substantially reduce aflatoxin contamination via direct replacement and additionally via direct contact or touch with toxigenic (Tox) isolates and secretion of inhibitory/degradative chemicals. To understand touch inhibition, HPLC analysis and RNA sequencing examined aflatoxin production and gene expression of Non-tox isolate 17 and Tox isolate 53 mono-cultures and during their interaction in co-culture. Aflatoxin production was reduced by 99.7% in 72 h co-cultures. Fewer than expected unique reads were assigned to Tox 53 during co-culture, indicating its growth and/or gene expression was inhibited in response to Non-tox 17. Predicted secreted proteins and genes involved in oxidation/reduction were enriched in Non-tox 17 and co-cultures compared to Tox 53. Five secondary metabolite (SM) gene clusters and kojic acid synthesis genes were upregulated in Non-tox 17 compared to Tox 53 and a few were further upregulated in co-cultures in response to touch. These results suggest Non-tox strains can inhibit growth and aflatoxin gene cluster expression in Tox strains through touch. Additionally, upregulation of other SM genes and redox genes during the biocontrol interaction demonstrates a potential role of inhibitory SMs and antioxidants as additional biocontrol mechanisms and deserves further exploration to improve biocontrol formulations.

## 1. Introduction

Aflatoxin is a deadly, acute and carcinogenic toxin to humans, livestock and wildlife [1,2,3,4,5]. Aflatoxin is produced by several different plant pathogenic fungi in *Aspergillus* section *Flavi* and contaminates corn, cottonseed, groundnuts and other oil-rich seeds [1,3,4,5]. *Aspergillus flavus* is blamed for most aflatoxin contamination events because it is most frequently isolated from affected grain [1,4,6]; however, closely related small sclerotia species including *A. agricola*, *A. texensis*, *A. toxicus*, *A. minisclerotigenes* and the Lethal Aflatoxicosis clade and more distant *A. parasiticus*, can also be isolated from crops and lead to aflatoxin contamination [7,8,9,10,11,12]. Aflatoxin contamination is especially common during hot and dry growing seasons [1,4]. Globally, aflatoxin is a major food concern and leads to deadly aflatoxicosis outbreaks in Africa [13,14]. It is estimated that aflatoxin contamination of corn costs the US between $50 million and $1 billion a year depending on the severity of the outbreak [2].

Currently, one of the most effective and widespread management tools to mitigate aflatoxin contamination is a pre-harvest biological control utilizing non-aflatoxigenic (Non-tox) isolates of *A. flavus* [15,16,17,18,19,20,21]. Sterilized grain coated with Non-tox *A. flavus* isolates are deployed on the soil surface in furrow to outcompete and overtake resident toxigenic (Tox) isolates both in/on the soil and crop. The first single-strain formulations of this type of biocontrol were developed for use on Arizona cotton (Af36 Prevail, Arizona Cotton Research and Protection Council, Phoenix, AZ, USA) and for use on Georgia peanuts (AflaGuard^®^, Syngenta Global, Basel, Switzerland) by scientists at the U.S. Department of Agriculture [17,18,19]. Now Non-tox biocontrol formulations are labeled for use on corn, almonds, pistachios and figs and recent research efforts are investigating the use in peppers [22]. Worldwide, biocontrol formulations are being developed and registered for use in Italy, Serbia, Argentina, and several African countries, including Nigeria, Kenya, Senegal, Gambia, Burkina Faso, Ghana, Tanzania, Mozambique, Malawi, and Zambia [16,20,23,24]. Many new formulations use multiple, locally-adapted Non-tox *A. flavus* strains, citing improved effectiveness over single-strain formulations [16,20,21,23,24].

The biocontrol is reported to competitively exclude Tox isolates primarily via direct replacement [17,25,26,27,28]; however, there are additional mechanisms that deserve further study [29,30,31]. When biocontrol is applied to soil surfaces, Non-tox isolate(s) germinate and produce copious conidia (asexual spores) [17,25,26,27,28]. Higher Non-tox inoculum load increases probability of Non-tox flower/seed infection and directly replaces or outcompetes the Tox [17,25,26,27,28]. Direct replacement with Non-tox results in substantial reduction in aflatoxin contamination [16,17,20,21,23,24,25,26,27]. Additionally, in both field and lab experiments, there is greater aflatoxin reduction than would be expected by a one-to-one replacement by Non-tox [15,32,33,34,35]. It is speculated the Non-tox outcompetes or occupies the niche faster, thereby excluding Tox isolates and there is an inhibition of aflatoxin production. Studies have shown that co-inoculation of Non-tox and Tox isolates on both artificial medium and corn, as Non-tox conidium abundance shifts from 20% to 80% [15,32,35], and relative abundance of Tox DNA to Non-tox DNA within kernels [33], the reduction in aflatoxin production is much more substantial than expected by direct replacement alone. This reduction in aflatoxin production is attributed to either plant responses to the Non-tox fungus [15,36] or interference from a different thallus preventing full colony development and delaying secondary metabolism [32]. Since separating Non-tox and Tox cultures by a 0.2 µm porous membrane does not alter aflatoxin production, but aflatoxin production decreased when pore sizes are larger than conidia and hyphae, it was hypothesized that direct contact between Non-tox and Tox isolates leads to an inhibition of aflatoxin production [34]. Recent evidence suggests that several other biocontrol Non-tox isolates and *Aspergillus oryzae* also produce diffusible chemicals that lead to a reduction in aflatoxin production [33,34,35,36,37,38,39,40]. Additionally, Non-tox isolates can degrade and use aflatoxin as a substrate [41]. The biocontrol may lower aflatoxin contamination by any number of possible mechanisms: directly replacing Tox with Non-tox, inhibiting toxin production by direct contract or touch, secreting diffusible inhibitory and/or degradative chemicals. However, it is still unclear exactly how the Non-tox isolates interfere with aflatoxin production.

Since little is known about how Non-tox isolates reduce aflatoxin production during the biocontrol interaction, an RNA-seq experiment was conducted to determine how gene expression of Tox and Non-tox isolates changed during co-cultivation. A highly inhibitory Non-tox isolate [39,40] from Louisiana was co-cultured with a widely distributed Tox isolate in Louisiana corn [42]. We present evidence of differences in expression of genes presumptively involved in oxidation/reduction reactions and production of proteins that are secreted outside the cell between Tox and Non-tox isolates. Additionally, expression of genes associated with secondary metabolite gene clusters was upregulated before and after contact between Tox and Non-tox isolates. We also present evidence that the Tox isolate grows less in the presence of the Non-tox isolate.

## 2. Results

RNA sequencing was conducted to better understand changes in gene expression during the biocontrol interaction between non-aflatoxigenic (Non-tox) and toxigenic (Tox) *Aspergillus flavus* isolates. During this in vitro interaction, aflatoxin production was inhibited. Tox isolate 53 and Non-tox isolate 17 were grown in mono-culture and together in co-cultures for 30 and 72 h, followed by aflatoxin extraction and quantification with HPLC, and total RNA extraction for mRNA library preparation and sequencing using Illumina NextSeq RNA sequencing technology.

### 2.1. Aflatoxin

Non-tox 17, Tox 53 and their co-cultures produced different quantities of aflatoxin B_1_ after growing in liquid medium for different time points (30, 72 and 96 h) as indicated by significant interactions (F_4,29_ = 207, *p*-value < 0.0001). Tox 53 started producing significant quantities of aflatoxin at 72 h of growth (Table 1). Very limited aflatoxin (<2 ppb) was detected in the biocontrol interaction samples consisting of Tox 53 and Non-tox 17 co-cultures, suggesting the presence of Non-tox 17 severely limited aflatoxin production by Tox 53. Additionally, aflatoxin degradation by Non-tox 17 may have resulted in lower aflatoxin [41], despite the addition of citrate buffer to limit aflatoxin degradation [39,40,43]. Non-tox 17 alone did not produce aflatoxin, thereby confirming its non-aflatoxigenic phenotype.

### 2.2. Fungal Biomass and Total RNA

**Figure 1:**
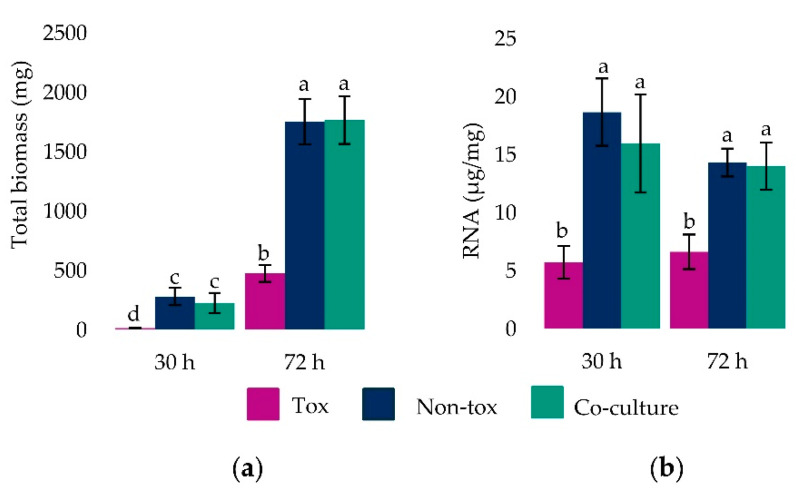
Mycelial biomass and RNA production by Tox 53 and Non-tox 17 alone, as well as during their biocontrol interaction in co-culture. Isolates were grown alone or in co-culture (i.e., biocontrol interaction) in 15 mL of liquid medium for 30 and 72 h. Mycelial tissue was harvested, (**a**) weighed and (**b**) then RNA was extracted. Mean ± SD from 5 reps at 30 h and 4 reps at 72 h. Different letters denote significance as per least squares means comparisons (α ≤ 0.05).

Tox 53, Non-tox 17 and their co-cultures produced different amounts of mycelial biomass at 30 and 72 h (F_2,21_ = 58.0, *p*-value < 0.0001). For each mono- and co-culture, there was more mycelial mass after 72 h (Figure 1). At both 30 and 72 h culture ages, Tox 53 produced less mycelia than Non-tox 17 and the co-cultures. Very little Tox 53 tissue was harvested at 30 h and the least squares estimate was not different from 0 (t_21_ = 0.38, *p*-value = 0.71). In contrast to the amount of mycelial tissue harvested, the differences between Non-tox 17, Tox 53 and their co-cultures in amount of total RNA extracted did not vary between 30 and 72 h (F_2,18_ = 1.82, *p*-value = 0.19) and culture age alone did not affect total RNA (F_1,18_ = 2.54, *p*-value = 0.13). This suggested there were no differences in RNA extraction efficiencies or in RNA production within older tissue. Isolate type affected the total RNA (F_2,18_ = 32.64, *p*-value < 0.0001) as less total RNA was extracted from Tox 53 than Non-Tox 17 and co-cultures.

### 2.3. RNA Sequencing

Between 11 and 30 million paired-end reads were sequenced for Non-tox 17, Tox 53 and co-cultures (Table 2). On average 37% of sequence reads mapped to genes with single-nucleotide differences between Non-tox 17 and Tox 53. In co-culture, only 3% of the unique reads were assigned to Tox 53, which would indicate very little presence or gene expression by Tox 53. That 3% was slightly more than 1% of reads misaligned to Tox 53 in Non-tox 17 pure cultures, suggested as little as 2% of the reads uniquely aligned to Tox 53 in co-culture.

#### 2.3.1. Equating Growth with RNA Production

The observed proportion of reads that uniquely aligned to Tox 53 in co-culture was very low (approximately 0.03 or 3%). Therefore, the observed proportion of Tox 53 reads in co-culture was compared to the expected proportions of Tox 53 based on differences in growth (biomass) or RNA production between Tox 53 and Non-tox 17. The expected proportion of Tox 53 biomass in co-culture was based on mono-cultures and calculated as:

p_53 biomass_ = (Tox 53 biomass (mg)) ÷ total biomass (Tox 53 biomass (mg) + Non-tox 17 biomass (mg))

**Figure 2:**
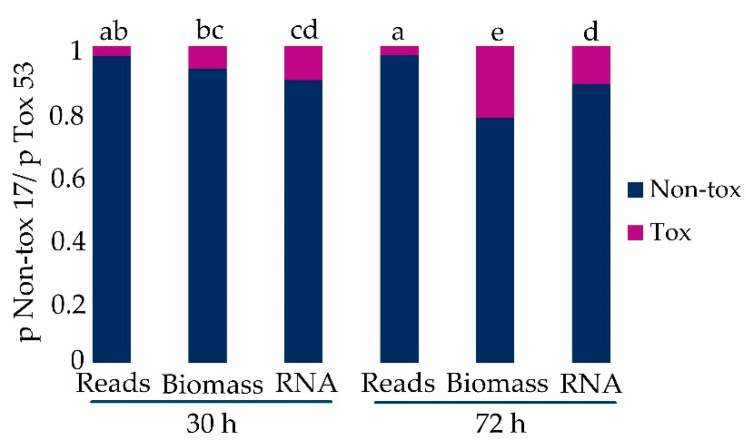
Proportion of RNA sequence reads uniquely aligned to *A. flavus* Tox 53 and Non-tox 17 in co-culture vs. the expected proportions based on biomass and RNA production of isolates grown apart. Proportions were compared using a generalized linear mixed model with the logit link and binomial distribution. Different letters denote significance as per least squares means (odds) comparisons (α < 0.05).

Total biomass was an estimate of co-cultures’ total biomass that assumed Tox 53 or Non-tox 17 do not influence the growth of either isolate. Expected proportion of Tox 53 was also calculated using RNA (µg/mg mycelium). Multicategorical data analysis (i.e., multiple contingency tables) compared the observed proportion of reads that uniquely aligned to Tox 53 in co-culture to the expected proportion based on Tox 53 biomass or RNA (Figure 2). There was a significant interaction between the proportion of Tox 53 as determined by reads, biomass, total RNA and 30 or 72 h time points (F_4,22_ = 9288, *p*-value < 0.0001). At 30 h, 3% of the reads were not significantly less than would be expected based on the growth of Tox 53, but at 72 h there were significantly fewer reads aligned to Tox 53 than would be expected based on both biomass and RNA production (Figure 2). This indicated that co-culturing Tox 53 with Non-tox 17 decreased both RNA transcription and growth of Tox 53. Growth medium was buffered with citrate to maintain pH~4 [39,40,43] and avoid acidification from fungal growth which reduces aflatoxin production and fungal growth, suggesting the reduced Tox 53 growth and transcription during co-culture is unlikely solely from acidification by Non-tox 17.

#### 2.3.2. Differential Gene Expression

**Figure 3:**
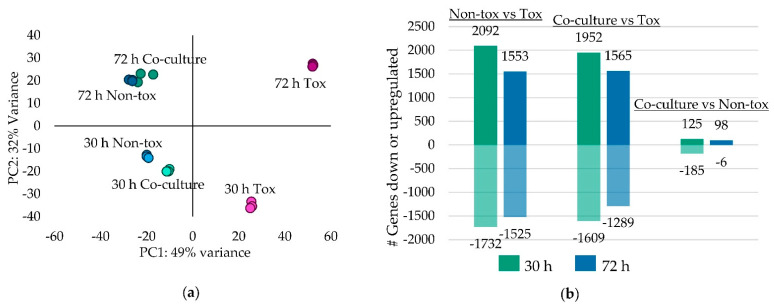
Differential gene expression of Tox 53, Non-tox 17 and their co-cultures. (**a**) Principal component analysis (PCA) of gene expression and (**b**) number of differentially expressed genes between Tox 53, Non-tox 17 and co-cultures. (**a**) PCA was generated for 500 genes with highest log regularized read count variance. Dots represent score for each biological replicate. (**b**) The number of upregulated genes are shown above the origin and the number of downregulated genes are shown below the origin.

Based on principle component analysis, the biological reps clustered closely together; however, 72 h co-cultures had the most variation (Figure 3a). Expression patterns differed between 30 and 72 h cultures and for each time point the co-cultures clustered closely with Non-tox 17. The Non-tox 17 mono-cultures and co-cultures expressed between 1500 and 2000 genes more than Tox 53 with similar amounts of gene downregulation in Tox 53 (Figure 3b). However, very few genes differed in expression between the co-cultures and Non-tox 17 mono-cultures because Non-tox 17 growth dominated Tox 53 growth in co-cultures.

#### 2.3.3. Functional Analysis of Differentially Expressed Genes

Like overall gene expression, gene ontology functional categories differed between Tox 53 and both Non-tox 17 and co-cultures, and very few categories differed between co-cultures and Non-tox 17, likely due to Non-tox 17′s growth dominating the co-cultures (Table 3). Hundreds of genes involved in oxidation/reduction reactions and encoding proteins localized to apoplastic or extracellular spaces were more differentially expressed than any of the other categories between Tox 53 and Non-tox 17. Genes that were typically upregulated in Non-tox 17 were also downregulated in Tox 53. Those trends were mostly consistent between co-cultures and Tox 53 as well. More than 50 zinc finger transcription factor genes were upregulated in Non-tox 17 and co-cultures compared to Tox 53. Most other gene categories were only differentially regulated at 30 or 72 h. Most of the secondary metabolite genes involved in aflatoxin, sterigmatocystin and cyclopiazonic acid production were all downregulated in Non-tox 17 and co-cultures compared to Tox 53. Non-tox 17 does not have any of the genes from these mycotoxin biosynthesis pathways. A few genes from these pathways were upregulated in co-cultures indicating there was some Tox 53 growth present; however, most pathway genes were not expressed at a detectable level. Genes from the biosynthesis pathways of the putative asperfuranone and characterized imizoquin secondary metabolites were also upregulated in Non-tox 17 compared to Tox 53.

#### 2.3.4. Gene Expression in the Aflatoxin Biosynthesis Cluster

The Non-tox 17 isolate does not have aflatoxin cluster genes, explaining the low expression levels indicated in Table 4. Some genes were expressed at a higher level in co-culture compared to Non-tox 17 alone, indicating limited growth of Tox 53 in co-culture. However, since there were fewer than 10 reads per gene at 72 h, there was very little expression of aflatoxin cluster genes, which is supported by the lack of aflatoxin production in co-culture. More genes were differentially expressed, and greater fold differences occurred at 72 h suggesting the lack of detectable aflatoxin at 30 h was due to very low expression of some aflatoxin cluster genes.

#### 2.3.5. Genes Highly Upregulated in Biocontrol Non-Tox 17 Compared to Tox 53

To understand what specific genes may contribute to Non-tox 17′s ability to outcompete Tox 53 and reduce its aflatoxin production during the biocontrol interaction, genes with the greatest upregulation (Log_2_-fold change ≥ 8) in Non-tox 17 compared to Tox 53 were selected (Table 5a). Differential gene expression between Non-tox 17 and Tox 53 was similar to that of co-culture and Tox 53 alone, likely due to limited growth of Tox 53 in co-culture. The upregulated genes in Non-tox 17 compared to Tox 53 represent a diversity of potential functions including oxidation/reduction reactions, peroxisome production, metabolism, and protein-protein interactions. These functions are consistent with the predominant gene functions identified by functional enrichment analysis. Interesting, most of the highly expressed genes were on chromosome 5. AFLA_060320 and AFLA_060350, and AFLA_095290 and AFLA_095300 were co-located, but there was no similar trend for surrounding genes to be differentially expressed in those regions of the chromosome (Table S1). However, nearby genes AFLA_062960, 062980, and 062990 were upregulated and are in a secondary metabolite cluster 20 as predicted by SMURF [44,45]. Most of the remaining genes in cluster 20 were also upregulated in both Non-tox 17 and co-cultures, suggesting a potential role for differential secondary metabolism during the biocontrol interaction (Table 5b). Gene cluster 20 is predicted to produce asperfuranone [45,46] which was only found to be enriched at 72 h. Here, the genes were all differentially expressed at 30 and 72 h. When Tox 53 grew alone, no reads aligned to genes in the latter portion of SMURF cluster 20, starting with AFLA_062940. Since only AFLA_062800-AFLA_062880 are required for asperfuranone production [46], the remaining genes in cluster 20 are likely a separate secondary metabolite cluster only produced by Non-tox 17.

#### 2.3.6. Genes Upregulated in Response to Tox 53

To further identify genes that may contribute to Non-tox 17′s ability to limit aflatoxin production during the biocontrol interaction, genes upregulated in response to Tox 53 were identified. Since co-cultures were dominated by Non-tox 17, genes that were further upregulated in Non-tox 17 in response to the presence of Tox 53 should have greater than 2 log_2_-fold differential expression in co-cultures than both Tox 53 and Non-tox 17 alone (Table S2). Only 10 genes fit these criteria and the fold changes only ranged from 2–3.2 when comparing co-cultures to Non-tox 17. If the criteria were loosened to greater than 1, 12 genes were upregulated compared to Non-tox 17 and Tox 53 in co-culture. Of the 12 genes, only AFLA_016350 (predicted NAD (P)H-dependent reductase) was expressed at a higher level in co-culture compared to Non-tox 17 at 30 h.

A closer inspection of the fold change values between co-cultures and Tox 53 revealed several genes that had slightly higher fold changes than Non-tox 17 alone vs. Tox 53, suggesting there could be a higher number of reads from Non-tox 17 in co-culture compared to Non-tox 17 alone, despite some relative expression levels indicating a lack of significance between co-culture and Non-tox 17. Reads per kilobase per millions of reads mapped (RPKM) values are shown for genes with higher RPKM values in co-culture than both Non-tox 17 and Tox 53 mono-cultures (Table 6a). Additionally, generalized linear models and post hoc least squares means (log odds) comparisons separated treatments based on normalized read counts per gene per total reads (proportion of total reads) (Table 6), like DESeq2 differential gene expression methodology on read counts without using their data smoothing algorithms [47]. Without smoothing an additional 17 genes, that were expressed at slightly higher levels in co-culture vs. Tox 53 comparisons than in the individual Non-tox 17 vs. Tox 53 comparisons, were found to have significantly more reads mapped to the co-culture than both Tox 53 and Non-tox 17 alone, suggesting that co-culturing the two isolates induced expression of several genes in Non-tox 17. Like differential expression using the fold changes from DESeq2, the greatest differential expression was at 72 h and most genes were expressed in larger abundance in co-cultures based on RPKM at 72 h.

Many genes induced in response to Tox 53 functions are membrane transport proteins, metal- or heme-binding proteins (potentially chelators), involved in oxidation/reduction reactions and metabolism. Several genes were part of a secondary metabolite cluster predicted by both antiSMASH [48] (cluster 8.5) and SMURF (cluster 46) [45], which encodes for the PKS responsible for orsellinic acid, a precursor to many polyketides like lecanoric acid and the pigments F-9775A and B [49] as well as meroterpenoids like LL-Z1272β (Ilicicolin B) [50]. The most highly-expressed genes in this cluster were the efflux pump and an O-methyl transferase that could convert the orsellinic acid precursor to 3,5-dimethylorsellicinc acid, itself a precursor to ausitinol in *A. nidulans* [49]. AFLA_096040 and AFLA_096060 are two of the three genes found in the kojic acid biosynthesis pathway [51]. Despite co-culture having a 1.3 log_2_-fold change from Non-tox 17 alone, AFLA_096040 (an oxidoreductase) had the greatest RPKM value of all genes upregulated in the co-culture, from both Non-tox 17 and Tox 53. The RPKM value for AFLA_096040 was 16X greater than AFLA_096060, suggesting that even though there was a smaller log_2_-fold change difference (1.3 vs. 2.2) between co-culture and Non-tox 17, there would be more mRNA molecules for AFLA_096040. Similarly, when comparing the RPKM values of highly upregulated genes in Non-tox 17 and co-culture compared to Tox 53 to RPKM values of genes upregulated in co-culture than both Tox 53 and Non-tox 17, only five of the 13 (38%) genes had RPKM values greater than 50 when selected based on greater than 8-fold changes (Table 6b). Conversely, 14 of the 29 (48%) genes had RPKM values greater than 50 despite low log_2_-fold changes (1–3.2) or lack of significant difference from DeSeq2 analysis between co-cultures and both Non-tox 17 and Tox 53. This suggests that when selecting influential genes, both abundance and relative abundance should be considered.

#### 2.3.7. Differential Expression of Imizoquin Biosynthesis Genes

Imizoquin biosynthesis was predicted to be enriched in Non-tox 17 compared to Tox 53; however, during close inspection of differential expression between Tox 53 and Non-tox 17 and co-cultures, none of the genes in imizoquin biosynthesis (*imq*) were highly differentially expressed (Table 3). Only 4 of 11 genes (AFLA_064230–064330) in the *imq* cluster [52] were found to be upregulated in both Non-tox 17 and co-cultures compared to Tox 53 at 72 h with log_2_-fold changes ranging between 1.8 and 4.8, (Table S1). However, upon comparing RPKM values there were differences in gene expression in between Tox 53, Non-tox 17 and co-cultures (Table 7). At both 30 and 72 h there was very little expression of genes in the *imq* cluster by Tox 53. However, it was found that at 30 h there is substantial expression of genes in Tox 53 from a secondary metabolite gene cluster (AntiSMASH cluster 1.1) adjacent to the *imq* cluster that may be associated with production of a toxic gliotoxin-like metabolite, likely aspirochlorine (AFLA_064340-AFLA_064610, *acl*) [53]. In several instances, there was less gene expression in co-cultures than Non-tox 17 though still greater than Tox 53, suggesting that imizoquin and aspirochlorine production is slightly attenuated in response to Tox 53.

## 3. Discussion

The current study investigated differences in gene expression during the biocontrol interaction between a non-aflatoxigenic *Aspergillus flavus* isolate (Non-tox 17) and a toxigenic isolate (Tox 53) which severely limits aflatoxin production by Tox 53. In support of the prevailing theory that competitive exclusion occurs by direct replacement of Tox with Non-tox isolates, Tox 53 grew much less both in mono-culture and co-culture with Non-tox 17. Since there was less Tox 53 RNA in co-cultures than expected based on its growth characteristics, in addition to significantly reduced transcription of both aflatoxin and CPA cluster genes, the data suggest Non-tox 17 is limiting gene expression and growth of Tox 53 as previously observed [32,33,37]. Non-tox 17 and other biocontrol isolates need to be further evaluated for their anti-fungal activity during co-culture. Limited growth of Tox 53 resulted in similar gene expression profiles between co-cultures and Non-tox 17. Expression of genes encoding proteins presumptively functioning in redox reactions, transcription factors and secreted proteins differed between Non-tox 17 and Tox 53 suggesting their possible roles in fungal growth and aflatoxin inhibition or degradation. Genes in select secondary metabolite clusters were either upregulated in Non-Tox 17 (asperfuranone and imizoquin) or further upregulated when co-cultured with Tox 53 (kojic acid and orsellinic acid). We are currently investigating if these secondary metabolites play a role in inhibition of aflatoxin production through both touch inhibition and recently reported contactless inhibition by chemicals secreted in culture filtrates from Non-tox (e.g., Non-tox 17) biocontrol isolates [37,38,39,40]. *Several genes with statistical differences between samples but a log_2_-fold change less than 2 had very high RPKM (>100–1000) values, whereas genes with the highest log_2_-fold changes had RPKM values typically under 50. This suggests that using log_2_-fold changes can* identify genes with high differential expression that are not expressed at high levels, therefore, RPKM values should also be considered to determine if differential expression of a gene will contribute more transcripts and potentially become more biologically influential. Based on our observations, biocontrol strains such as Non-tox 17 likely lower aflatoxin contamination by a combination of outcompeting and displacing Tox 53 and producing secondary metabolites, which may alter the redox state and extracellular environment or otherwise inhibit important cellular processes.

The majority of differentially expressed genes in the Non-tox 17 mono-culture and during co-culture were involved in oxidation and reduction reactions. It is hypothesized that aflatoxin is produced to minimize oxidative stress from the host plant’s oxidative burst that occurs during fungal invasion or drought stress [36,54,55]. Several genes in the aflatoxin biosynthesis pathway are sources of reactive oxygen species (ROS) [54] and mutants and natural non-aflatoxigenic *A. flavus* and *A. parasiticus* strains are more sensitive to growth medium amended with H_2_O_2_ [54,55]. Aflatoxin production is induced by H_2_O_2_ and it was suggested that during aflatoxin synthesis, antioxidative enzymes scavenge H_2_O_2_ from the environment and sequester ROS in vesicles, thereby alleviating oxidative stress in the fungus [54,55,56]. Alternatively, aflatoxin production may be a source of oxidative stress to the fungus due to a buildup of ROS, and it was shown that toxigenic isolates have greater glutathione S-transferase activity at the onset of aflatoxin production in comparison with Non-tox isolates [57,58]. Glutathione S-transferase activity should mollify oxidative stress resulting in a decrease in aflatoxin production [57,58]. Interestingly, most corn isolates are Non-tox or low toxin producers [42], provide the majority of biomass during co-infection of kernels with Tox isolates [33], and survive greater ROS defense responses from plants [36]. This suggests Non-tox isolates have alternative mechanisms to alleviate oxidative stress which may explain why we observed that most differentially expressed genes are involved in oxidation and reduction reactions. NRRL 21882, the Non-tox isolate in AflaGuard^®^, differentially expressed more genes involved in oxidation and reduction than Tox isolates from H_2_O_2_–induced oxidative stress [56]. Further studies are needed to determine if Non-tox isolates alter the redox environment, resulting in decreased aflatoxin production and invasion of plant tissue by Tox isolates.

In addition to limited growth of Tox 53 during co-culture with Non-tox 17, there was also reduced expression of aflatoxin biosynthesis pathway genes. Multiple Non-tox isolates downregulated *aflR*, *aflJ*, *omtA*, *ordA*, *pksA*, and *vbs* when co-cultured with Tox isolates [59]. During co-culture, it is impossible to rule out that inhibition of aflatoxin production is only due to outcompeting the Tox isolate by the Non-tox isolate since here Tox 53 grew substantially less than Non-tox 17. However, cell-free Non-tox media filtrates from *A. flavus*, including Non-tox 17 and *A. oryzae*, inhibited aflatoxin production [37,38,39,40,60] or degraded aflatoxin [41]. Genes in the early and middle portions of the aflatoxin biosynthesis pathway were downregulated in NRRL 3357 in response to A. *oryzae* filtrates [60]. The aflatoxin biosynthetic pathway-specific co-activator, *aflS*, was substantially downregulated, but there was not significantly less expression of the transcriptional activator *aflR* [60]. Contrary to our findings, there was greater expression of imizoquins and cyclopiazonic acid upon exposure to only culture filtrates [60]. These results indicate that Non-tox isolates may lower aflatoxin production by both displacement and inhibition of aflatoxin production through production of chemicals capable of downregulating expression of critical aflatoxin biosynthetic pathway genes.

Expression of several secondary metabolite cluster genes was either upregulated more in Non-tox 17 compared to Tox 53 and/or further upregulated in response to Tox 53 during co-culture. Some of these may be candidate compounds that interfere with aflatoxin production during the biocontrol interaction. Genes involved in kojic acid synthesis had the greatest RPKM values during co-culture. Kojic acid is commonly found in soy sauce and miso, and functions as an antioxidant that inhibits browning due to polyphenol oxidases in potatoes, apples and mushrooms [61]. It is also used in the cosmetic industry to lighten skin by inhibiting melanization [61]. During the biocontrol interaction, kojic acid may serve as an antioxidant resulting in less aflatoxin production by Tox isolates. Under elevated H_2_O_2_–induced oxidative stress, *kojA* expression increased in NRRL 3357 and NRRL 21,882 (AflaGuard), while other Tox and Non-tox isolates demonstrated normal levels of *kojA* expression [56]. In this manuscript, 30 and 72 h Non-tox 17 fungal cultures produced more transcripts than one-week-old cultures in Fountain et al. [56], suggesting transcription of genes in kojic acid synthesis may diminish with culture age, or Non-tox 17 produces much more kojic acid transcripts than other *A. flavus* isolates. Although the RPKM values were less, genes in the predicted orsellinic acid biosynthesis cluster (antiSMASH cluster 8.5, SMURF 46) [45] were also upregulated in response to Tox 53. The orsellinic acid gene in *A. nidulans* was turned on when the fungus physically interacted with the bacterium *Streptomyces rapamycinicus* [62], resulting in production of orsellinic acid and its derivatives: lecanoric acid, F-9775A, and F-9775B. A similar phenomenon could be occurring in our experiments (e.g., increased expression of the orsellinic acid biosynthesis cluster genes occurring after contact with other fungal hyphae). Predicted asperfuranone genes (SMURF cluster 20) [45,46] were expressed more by Non-tox 17 and co-cultures than Tox 53. Asperfuranone inhibits growth of small lung cancer cells and induces apoptosis [63], suggesting that asperfuranone could potentially inhibit growth of Tox 53. Finally, imizoquin cluster genes [52] were expressed at higher levels by Non-tox 17 at 30 and 72 h compared to Tox 53; co-cultures expressed intermediate levels. Imizoquins were downregulated in response to an isolate of *Ralstonia solanacearum* that produced a lipopeptide, which induced chlamydospore production in *A. flavus* [52,64]. Loss of imizoquin production delays spore germination and increases sensitivity to H_2_O_2_–induced oxidative stress [52] suggesting it is involved in spore germination and can act as an antioxidant. Continued expression of imizoquin cluster genes by Non-tox 17 may reduce aflatoxin production in Tox 53 by reducing oxidative stress. Future metabolomic studies will be used (1) to determine if kojic acid, orsellinic acid, asperfuranone, and imizoquins are produced by Non-tox 17 alone and in co-culture, and (2) to understand how they regulate growth and aflatoxin production of *A. flavus*.

Non-tox *A. flavus* isolates are widely used as biocontrol agents to effectively manage aflatoxin contamination of peanuts, corn, cottonseed and pistachios [15,16,17,18,19,20,21]. Although the biocontrol has been shown to work primarily via direct replacement of Tox isolates with Non-tox isolates [17,25,26,27,28], as was confirmed in this manuscript, it is important to understand how Non-tox isolates molecularly and biochemically inhibit growth and toxin production of Tox *A. flavus*. Secondary metabolites previously found to be regulated in response to other microorganisms also produced different numbers of transcripts. Kojic acid and imizoquins, along with different individual genes, potentially alter aflatoxin production by serving as antioxidants. The greater antioxidant activity provided by kojic acid, imizoquins and other oxidation/reduction genes potentially gives the Non-tox a competitive advantage when infecting crops. Asperfuranone potentially acts in the biocontrol interaction by inhibiting growth. Future directions include determining if these chemicals are produced during the biocontrol interaction and assess their effects on *A. flavus* growth. If *A. flavus* chemicals (i.e., secondary metabolites) inhibit aflatoxin production, biocontrols should be evaluated for production of the most inhibitory chemicals, and then engineered to overproduce those chemicals or developed into a spray treatment mimicking the presence of Non-tox *A. flavus*.

## 4. Materials and Methods

### 4.1. Fungal Isolates

*Aspergillus flavus* Non-tox isolate 17, also named 07-S-3-1-6 (SRRC1588), was isolated from Louisiana corn field soil in 2007 [42] and is highly inhibitory to aflatoxin production [39,40]. Tox isolate 53 (SRRC1669) was isolated from Louisiana-grown, surface-sterilized corn kernels in 2003 [34], is highly toxigenic, and belongs to vegetative compatibility group RRS4 [42] originally isolated from corn kernels throughout Louisiana and along the Mississippi River in the US [65]. Tox 53 demonstrated the importance of physical interaction for toxin inhibition during a previous biocontrol interaction [34]. Both isolates are deposited in an accessible culture collection at the USDA-ARS Southern Regional Research Center (SRRC) in New Orleans Louisiana.

### 4.2. Biocontrol Interaction Cultural Experimental Design

RNA and aflatoxin were extracted from Tox 53 and Non-tox 17 isolates grown alone in mono-culture and together in co-cultures to understand how their gene expressions and aflatoxin production change in response to one another during the biocontrol interaction (i.e., co-cultures). Fresh conidia from 5-day old Tox 53 and Non-tox 17 cultures (grown in dark at 30 °C on 5% V8 juice, 2% agar, pH 5.2 medium) were suspended in glucose salts liquid medium (GS), with citrate buffer (pH 4) [32,34,43,66], at 1 × 10^5^ conidia/mL. Citrate buffer was added to maximize aflatoxin production, limit aflatoxin degradation and limit detrimental effects to fungal growth by low pH [39,40,43,66]. Buffered GS medium consisted of one part water, 2 parts 2.5 X salts and 2 parts 2.5 X glucose, mixed after autoclaving. One liter of 2.5 X salts consisted of 8.76 g (NH_4_)SO_4_, 1.88 g KH_2_PO_4_, 0.88 g MgSO_4_·7H_2_O, 0.188 g CaCl_2_·2H_2_O, 0.025 g ZnSO_4_·7H_2_O, 0.0125 g MnCl_2_·4H_2_O, 0.005 g (NH_4_)_6_Mo_7_O_24_·4H_2_0, 0.005 g Na_2_B_4_O_7_·10H_2_O and 0.005 g FeSO_4_·7H_2_O with 25.36 g citric acid and 17.03 g sodium citrate. One liter of 2.5 X glucose consisted of 125 g glucose. For mono-cultures, 15 mL of Non-tox 17 or Tox 53 conidial- GS medium suspensions were dispensed into 100 × 15 mm Petri dishes. For co-cultures, equal volumes of Non-tox 17 and Tox 53 conidial- GS medium suspensions were mixed and 15 mL were dispensed into the Petri dishes. Separate cultures were grown for 30, 72 and 96 h. There were at least 4 biological replicates for each cultural condition (Table 8). To obtain enough tissue for RNA extraction at 30 h, a biological replicate consisted of 9 Petri-dish cultures. For all other time points, only one Petri-dish was used per biological replicate. The isolate mono-cultures, co-cultures and time points grew in separate boxes (five Petri dishes per box) within a darkened 25 °C incubator to minimize potential effect of differential volatile production between isolates on aflatoxin production [66,67]. RNA was extracted from 30 and 72 h cultures and aflatoxin was extracted from 30, 72 and 96 h cultures.

### 4.3. Aflatoxin Extraction and Quantification

At 30 h, 100 µL of liquid medium from each dish (*n* = 9) per biological rep was added to HPLC grade methanol. At 72 and 96 h, 500 µL of medium from each biological replicate (a single Petri dish) was added to 500 µL of HPLC grade methanol. Extracts were filtered through 200 mg basic alumina (58Å, 60-mesh powder, 11503-A1, Alfa Aesar, Tewksbury MA) in 1.5 mL polypropylene columns with 20 µm polyethylene frits [68]. 10 µL of each sample was separated in a Waters e2695 Separations Model HPLC (Waters Corp., Milford, MA) using a Nova-Pak C18 4 µm, 3.9 mm × 150 mm column held at 38 °C with an isocratic solvent system (37.5 Methanol: 62.5 water at a 0.8 mL/min) coupled to a PHRED photochemical reactor cell (Aura Industries Inc., New York, NY, USA). After separation and photolytic derivatization, a 2475 FLR Detector (Waters Corp., Milford, MA, USA) was used to detect and quantify aflatoxin B_1_ (365 nm Ex, 440 nm Em) [69,70]. Run time was 17 min with aflatoxin B_1_ peak eluting at~13.5 min. Empower software (Waters Corp., Milford, MA, USA) was used to integrate the aflatoxin B_1_ peak. Aflatoxin quantity was calculated based on a calibration curve calculated from 4 replicates of standards with 1, 5, 50, 500 and 1000 ng/mL aflatoxin B_1_ [70]. Aflatoxin B_1_ minimum level of detection was <0.05 ppb and minimum quantification from standard curve was 1 ppb.

### 4.4. Whole Fungal Mycelia Harvest and RNA Extraction

At 30 and 72 h, mycelia and medium were removed from the Petri dishes and centrifuged at 8000× *g* for 5 min at 4 °C. Thirty-hour tissues from nine plates per biological rep were pooled and centrifuged a second time for 5 min. Excess medium was removed by carefully blotting mycelia on chromatography paper. The tissue was added to a pre-weighed microcentrifuge tube (to calculate wet weight) and flash frozen with liquid nitrogen. RNA extraction was performed according the manufacture’s guidelines for the Spectrum™ Plant Total RNA Kit (STRN250, Sigma-Aldrich, St. Louis, MO, USA) and the On-column Dnase I Digestion Set (DNASE70, Sigma-Aldrich, St. Louis, MO, USA) with a couple of modifications. All tissue from a single biological replicate was ground directly in lysis buffer (100 mg mycelia/500 μL lysis buffer). A few 30 h cultures had less than 100 mg, which were still ground in 500 μL lysis buffer. For each sample, 500 μL was retained for RNA extraction. Binding buffer was increased to 750 μL due to inefficient RNA extraction from the residual medium.

### 4.5. RNA Sequencing and Analysis

Three RNA extracts per experimental condition were sequenced by NC State University’s Genomic Sciences Laboratory using an Illumina NextSeq 500, which generated 150 bp paired-end reads. Sequencing reads were submitted to NCBI’s Sequence Read Archive and can be accessed under BioProject ID PRJNA764255. Sequence reads were trimmed to remove adapters and low-quality sequences using BBDuk [71]. Sequencing reads were mapped to the *A. flavus* NRRL 3357 genome (JCVI-afl1-v2.0 assembly, (https://www.ncbi.nlm.nih.gov/assembly/GCF_000006275.2/#/st, accessed on 8 April 2019) using STAR v2.6.1 [72]. Reads mapped to exons were counted using featureCounts v1.6.0 [73] followed by differential expression testing of normalized reads using a generalized linear model with log link and a negative binomial distribution within DESeq2 [47]. Genes were removed if they did not have at least 10 reads in 3 or more samples. Genes were considered differentially expressed if the pairwise comparison by DESeq2 software *p*-value was less than 0.05 and if there was a log_2_-fold change greater than 2 [47]. To make the principal component analysis (PCA) plot, regularized log counts were produced with the DESeq2′s rlog function and the option “blind = TRUE” was set [47]. These were used as input to the plotPCA function in DESeq2 [47]. In order to quantify the fraction of RNA-seq reads contributed by each strain, variants were called using Freebayes [74]. Variants that were different between Non-tox 17 and Tox 53 were used in a custom python script utilizing the pysam library (https://github.com/pysam-developers/pysam, accessed on 8 April 2019)) and SAMtools [75] to assign reads from the mixed cultures to each strain. Functional enrichment analysis was performed with the enrichment function in BC3NET R package [76], which uses a one-sided Fisher’s Exact test with the Benjamini and Hochberg adjustment [77].

Excel version 2102 (Microsoft corp., Redmond, WA) was used to sort pairwise log_2_-fold differential gene expression testing from DESeq2 for each pairwise comparison of Non-tox 17 vs. Tox 53, Co-culture vs. Tox 53, and Co-culture vs. Non-tox 17 at 30 and 72 h. Genes that were overexpressed in biocontrol isolate Non-tox 17 were selected if the log_2_-fold change was ≥8. Genes that were further upregulated in Non-tox 17 during co-culture were selected if Co-culture vs. Tox 53 and Co-culture vs. Non-tox 17 log_2_-fold changes were >1. Additionally, further upregulated genes were selected if the differences between Co-culture vs. Tox 53 and Non-tox 17 vs. Tox 53 were log_2_-fold changes at least 1. Since the latter selection criterion was not statistically different based on DESeq2 analysis of normalized reads, generalized linear models were calculated to compare gene expression for each of those genes using the logit (log odds, i.e., (proportion reads (proportion (p) reads aligned to gene X/(p reads not aligned to gene X)) link for binomial data with SAS version 9.4 (SAS Institute, Cary, North Carolina). The fixed effects were culture type (Non-tox 17, Tox 53 and Co-culture) and culture age (30 and 72 h). The response variable was reads/total reads. Treatments were separated by post hoc comparison of odds with a difference of least squares means at α ≤ 0.05. Excel was also used to calculate reads per kilobase per million mapped reads (RPKM) for genes selected by sorting. RPKM for gene X = (1 × 10^9^) (read mapped to gene X)/(gene X length bp) (total reads mapped) [47,78].

### 4.6. Other Data Analysis

Generalized linear models estimated multivariate analysis of variance to compare biomass, total RNA and aflatoxin B_1_ between treatments using SAS. To address issues with normality, aflatoxin values were log transformed. In each model, fixed effects were either isolate growing alone or in co-culture, extraction time, and their interaction. Means were separated by post hoc comparison with a difference of least squares means at α ≤ 0.05. To determine if the number of reads which uniquely aligned to Non-tox 17 and Tox 53 during co-culture was similar to the expected ratio based on biomass and RNA production of each isolate growing separately, generalized linear models estimated multiple categorical data analysis (i.e., multiple contingency tables) using logit link and binomial distribution with SAS. Log odds (p Tox 53/p Non-Tox 17) were calculated within the model by inputting the events (either number of unique reads, biomass or total RNA of the Non-tox) and dividing by trials (total number of reads, sum of biomass and total RNA of Non-Tox 17 and Tox 53 isolates). Odds were separated by post hoc comparison with a difference of least squares means at α ≤ 0.05.

## Figures and Tables

**Table 1:** Aflatoxin B_1_ production by Tox 53 and Non-tox 17 isolates alone and during biocontrol interaction in co-cultures.

	30 h	72 h	96 h
Cultures	Aflatoxin B_1_ ppb ± S. D.		
Tox 53	<0.05 c ^1^	680 ± 35 b	1902 ± 163 a
Non-tox 17	<0.05 c	<0.05 c	<0.05 c
Co-culture	0.2 ± 0.1 c	1.8 ± 0.2 c	<0.05 c

^1^ Mean ± SD from 5 reps at 30 h and 4 reps at 72 and 96 h. Aflatoxin B_1_ minimum level of detection by HPLC was <0.05 ppb and minimum quantification from standard curve was 1 ppb. Aflatoxin values with different letters denote significance as per least squares means comparisons (α ≤ 0.05).

**Table 2:** Total sequence reads (M) and reads (M) uniquely aligned to Tox 53 or Non-tox 17.

	30 h					72 h				
	Rep ^1^	Total ^2^	Non-Tox ^3^	Tox ^4^	P Tox ^5^	Rep ^1^	Total ^2^	Non-Tox ^3^	Tox ^4^	P Tox ^5^
Tox 53	a	14.2	0.092	4.336	0.979	d	8.6	0.050	2.985	0.984
	b	17.4	0.105	5.143	0.980	e	15.9	0.087	5.484	0.984
	c	14.3	0.085	4.206	0.980	f	14.1	0.105	4.817	0.979
Non-tox 17	a	16.7	5.107	0.082	0.016	d	16.9	5.819	0.085	0.014
	b	16.6	5.047	0.084	0.016	e	14.7	5.075	0.075	0.015
	c	18	5.358	0.090	0.017	f	14.2	5.040	0.074	0.014
Co-culture	a	29.6	8.267	0.299	0.035	d	14.0	4.891	0.140	0.028
	b	12.5	3.902	0.127	0.032	e	15.1	5.220	0.181	0.033
	c	11.3	3.510	0.120	0.033	f	29.1	9.497	0.261	0.027

^1^ RNA was sequenced from three independent replicates of Tox 53, Non-tox 17 mono and co-cultures. RNA was sequenced from different cultures at 30 and 72 h. ^2^ Total millions (M)-of 150 bp paired-end reads from Illumina RNA sequencing. ^3^ Millions (M) of reads uniquely aligned to Non-tox 17 based on single-nucleotide polymorphisms (SNPs) between Tox 53 and Non-tox 17. ^4^ Millions (M) of reads uniquely aligned to Tox 53 based on single-nucleotide polymorphisms SNPs between Tox 53 and Non-tox 17. ^5^ Proportion of reads that uniquely align to Tox 53 vs. Non-tox 17.

**Table 3:** Number of differentially expressed genes within significantly enriched functional annotation terms in Tox 53, Non-tox 17 and their co-cultures.

		30 h						72 h					
Functional Annotation Terms ^1^	Genes ^2^	Non vs. Tox ^3^		Co vs. Tox		Co vs. Non		Non vs. Tox		Co vs. Tox		Co vs. Non	
Oxidation/reduction	1477	274	-229		−235		−43	277		284			
Signal peptide	1188	236	−182	221	−176	28		220		212			
Extracellular	876		−146		−140	21		180		175			
Apoplastic	537	114	−93		−91	19		121		119			
NAD(P)-binding	596		−104		−118			114		124			
Oxidoreductase activity	542		−94	76	−95			102		113			
Major facilitator family	389	87		89									
Alpha/Beta hydrolase	313							85		81			
Zn(2)-C6 transcr. factor	289	67		69				57		57			
Iron ion binding	266	64		58									
FAD/NAD(P)-binding	289						−13	55		56			
Heme binding	246							48		50			
Monooxygenase activity	214						−12	46		49			
FAD binding	179							42		37			
Cytochrome P450	166							40		41			
GroES-like	117		−30		−33								
Fatty acid biosynthesis	99		−26		−24								
Alcohol dehydrogenase	93		−26		−29								
Peroxisome	71		−25		−23								
Polyketide synthase	94		−24		−28								
Tyrosine metabolism	71							23		20			
ATPase movement	66							18		19			
Isomerase activity	56		−19		−20								
AMP-binding site	57		−18										
Phenylalanine metab.	42							15		14			
Aflatoxin synth. cluster	29		−14		−11	16			−28		−28	9	
Obsolete peroxidase rxn	34		−14		−14								
Sterigmatocystin synth.	25					7			−14		−13	5	
Imizoquin synth.	11	7					−6	11		11			
Crotonase superfamily	19		−9		−11								
L-phenylalanine metab.	23							10					
Styrene catabolism	20							10		9			
Aromatic amino acid	20							10		10			
Phosphorelay sensor	20								−10		−8		
Protein histidine kinase	20								−10		−8		
Disulphide reductase	10							8		9			
Enoyl-CoA hydratase	9		−7		−7								
Sulfatase, conserved site	8	6		6				5					
Asperfuranone synth.	5							5		5			
Mycotoxin biosynthesis	4							4		4			
Cyclopiazonic acid	4		−4		−4	3			−4		−4	2	
Haem peroxidase	4		−4		−4								

^1^ Within each functional annotation term are. ^2^ Total number of genes assigned to the category. ^3^ Number of genes that were up and down (-) regulated in pair-wise comparisons between Non-tox 17 versus (v) Tox 53, co-culture (i.e., biocontrol interaction) vs. Tox 53, and co-culture vs. Non-tox 17 at 30 h and 72 h if the Benjamini and Hochberg adjusted *p*-value for the enrichment test was ≤ 0.05. Cells without numbers were not significantly enriched at α < 0.05. Values are color-scaled, blue is less than zero and red is greater than zero. A darker shade indicates a more negative or positive value and scaled to the maximum and minimum values in table.

**Table 4:** Differential expression of aflatoxin cluster genes between Non-tox 17, Tox 53 and their co-cultures.

		30 h ^1^			72 h			
Gene ID ^2^	Chr ^3^	Non vs. Tox	Co vs. Tox	Co vs. Non	Non vs. Tox	Co vs. Tox	Co vs. Non	Gene Synonyms or Putative Function
AFLA_139150	3	-	-	-	−15.1	−11.3	-	*aflY/hypA/hypP*
AFLA_139160	3	-	-	-	−14.8	−10.8	-	*aflX/ordB*
AFLA_139170	3	-	5.1	5.1	−16.1	−11.3	-	*aflW/moxY*
AFLA_139180	3	-	-	5.3	−15.2	−10.7	-	*aflV/cypX*
AFLA_139190	3	-	4.4	6.8	−15.3	−10.9	-	*aflK/vbs/verB*
AFLA_139200	3	-	-	-	−14.3	−10.9	-	*aflQ/ordA/ord-1*
AFLA_139210	3	-	-	-	−15.5	−10.7	-	*aflP/omtA/omt-1*
AFLA_139220	3	-	-	-	−15.2	−9.9	-	*aflO/omtB/dmtA*
AFLA_139230	3	-	-	-	−9.8	−9.1	-	*aflI/avfA*
AFLA_139240	3	-	-	-	-	-	-	
AFLA_139250	3	-	-	-	−15.3	−11.0	-	*aflL/verB*
AFLA_139260	3	-	-	-	−15.1	−10.2	-	*aflG/avnA/ord-1*
AFLA_139270	3	−11.3	−5.3	6.0	−16.1	−9.1	7.0	
AFLA_139280	3	-	-	4.4	−14.2	−9.8	-	*aflN/verA*
AFLA_139290	3	-	-	-	−11.9	−12.1	-	*aflMa/hypE*
AFLA_139300	3	-	3.0	3.5	−17.0	−10.5	6.5	*aflM/ver-1*
AFLA_139310	3	−5.9	-	5.9	−16.0	−11.9	-	*aflE/norA/aad/adh-2*
AFLA_139320	3	−9.6	−4.7	4.9	−15.7	−10.5	5.2	*aflJ/estA*
AFLA_139330	3	−9.9	−4.0	5.9	−16.0	−10.7	-	*aflH/adhA*
AFLA_139340	3	−11.6	−4.9	6.7	−15.0	−9.8	5.3	*aflS*
AFLA_139360	3	−11.6	−5.1	6.6	−11.8	−7.9	-	*aflR/apa-2/afl-2*
AFLA_139370	3	−10.2	−4.1	6.1	−15.1	−10.3	4.7	*aflB/fas-1*
AFLA_139380	3	−9.2	−3.6	5.6	−14.7	−10.4	4.2	*aflA/fas-2/hexA*
AFLA_139390	3	−8.1	−2.5	5.5	−15.8	−11.2	4.5	*aflD/nor-1*
AFLA_139400	3	−5.6	−3.2	-	−15.0	−10.9	-	*aflCa/hypC*
AFLA_139410	3	−8.2	−4.1	4.1	−16.0	−10.5	5.5	*aflC/pksA/pksL1*
AFLA_139420	3	−8.2	−3.1	5.0	−14.4	−8.7	5.7	*aflT/aflT*
AFLA_139430	3	−4.0	-	-	−11.2	−9.1	-	*aflU/cypA*
AFLA_139440	3	−3.5	-	-	−10.2	−8.6	-	*aflF/norB*
AFLA_139450	3	-	-	-	-	-	-	
AFLA_139460	3	−9.3	−5.7	-	−14.2	−9.7	-	MFS multidrug transporter
AFLA_139470	3	−14.3	−7.2	7.1	−13.1	−6.3	6.8	FAD-dependent oxidoreductase
AFLA_139480	3	−12.4	−6.4	6.0	−12.7	−5.5	7.2	Tryptophan synthase
AFLA_139490	3	−11.9	−6.7	5.2	−9.9	−7.0	-	hybrid PKS/NRPS enzyme

^1^ Log_2_-fold changes for gene expression pair-wise comparisons in Non-tox 17 versus (v) Tox 53, Co-culture vs. Tox 53, and Co-culture vs. Non-tox 17 at 30 and 72 h if the fold change was ≥2 and *p*-values were ≤0.05. A dash is reported for comparisons not meeting this criteria. Values are color-scaled, blue is less than zero and red is greater than zero. A darker shade indicates a more negative or positive value and scaled to the maximum and minimum values in table. ^2^ Gene ID is the AFLA_# gene number designated in the *A. flavus* NRRL 3357 reference genome. ^3^ Chr is the chromosome where aflatoxin cluster genes are located.

**Table 5:** Genes highly upregulated in Non-tox 17 mono-cultures and co-cultures compared to Tox 53 mono-cultures and differential gene expression in SMURF secondary metabolite cluster 20 (asperfuranone).

		30 h ^1^			72 h				
Gene ID ^2^	Chr ^3^	Non vs. Tox	Co vs. Tox	Co vs. Non	Non vs. Tox	Co vs. Tox	Co vs. Non	SM ^4^	Putative Function
(**a**) Genes with at least 8 log_2_-fold differential expression									
AFLA_085640	1	8.4	8.0	-	4.7	4.8	-	-, -	Peroxisome biogenesis
AFLA_025220	2	11.9	11.4	-	7.8	7.8	-	-, -	UPF0047 protein family
AFLA_126420	2	7.9	8.5	-	9.6	9.4	-	-, -	Protein glycosylation
AFLA_060320	5	8.6	8.4	-	7.3	7.1	-	-, -	Perforin domain
AFLA_060350	5	9.0	8.5	-	6.1	6.5	-	-, -	Unknown
AFLA_062960	5	10.1	10.2	-	10.3	10.2	-	-, 20	Zn(2)-C6 transcript. factor
AFLA_062980	5	9.8	10.0	-	9.8	9.7	-	-, 20	Crotonase activity
AFLA_062990	5	8.7	8.7	-	8.4	8.3	-	-, 20	Oxidase
AFLA_095290	5	9.5	9.6	-	2.3	2.9	-	-, -	Unknown
AFLA_095300	5	8.4	8.3	-	2.6	3.0	-	-, -	Protein-protein interactions
AFLA_095800	5	7.9	8.0	-	6.1	5.7	-	-, -	Short-chain reductase
AFLA_066370	6	8.9	8.7	-	8.7	8.8	-	-, -	Phosphorylation
AFLA_008080	8	10.4	9.6	-	-	-	-	-, -	Unknown
AFLA_117340	8	6.4	8.0	-	7.9	-	-	-, -	2-methylcitrate catabolism
(**b**) Differential gene expression in SMURF-predicted secondary metabolite cluster 20 (asperfuranone)									
AFLA_062800	5	2.4	2.4	-	1.9	1.9	-	-, 20	Aldo-keto reductase
AFLA_062810	5	3.6	3.6	-	5.1	4.9	-	-, 20	Hypothetical protein
AFLA_062820	5	3.3	4.5	1.2	5.3	5.0	-	-, 20	Polyketide synthase
AFLA_062830	5	3.8	4.2	-	5.0	4.8	-	-, 20	Monoxy./oxidoreductase
AFLA_062840	5	2.2	2.3	-	2.9	2.7	-	-, 20	Serine hydrolase
AFLA_062850	5	3.2	2.9	-	-	-	-	-, 20	Fatty acid oxidoreductase
AFLA_062860	5	3.5	4.4	-	5.4	5.1	-	-, 20	Polyketide synthase
AFLA_062890	5	1.2	-	-	1.6	1.2	-	-, 20	Hypothetical protein
AFLA_062940	5	7.4	7.5	-	6.6	6.6	-	-, 20	Choline transport protein
AFLA_062950	5	4.8	4.8	-	5.1	4.8	-	-, 20	Hypothetical protein
AFLA_062960	5	10.1	10.2	-	10.3	10.2	-	-, 20	Zn(2)-C6 transcript. factor
AFLA_062970	5	3.8	4.9	-	4.9	5.0	-	-, 20	Copper oxidase
AFLA_062980	5	9.8	10.0	-	9.8	9.7	-	-, 20	Crotonase activity
AFLA_062990	5	8.7	8.7	-	8.4	8.3	-	-, 20	Iron-binding oxidase
AFLA_063000	5	5.4	5.3	-	6.2	5.7	-	-, 20	Metal-binding hydrolase
ALFA_063020	5	4.3	5.1	-	5.4	6.0	-	-, 20	Multidrug resistance pump

^1^ Log_2_-fold changes for gene expression pair-wise comparisons of Non-tox 17 versus (v) Tox 53, Co-culture vs. Tox 53, and Co-culture vs. Non-tox 17 at 30 and 72 h if the fold change was ≥2 and *p*-values were ≤0.05. A dash is reported for comparisons not meeting these criteria. Values are color-scaled with a darker red tint indicating a more positive value and scaled to the maximum value in table. ^2^ Gene ID is the AFLA_# gene number designated in the *A. flavus* NRRL 3357 reference genome. Gene ID fonts color-coded in blue, red or gray are in close proximity on chromosomes. ^3^ Chr is the chromosome where each gene is located. ^4^ SM is the secondary metabolite cluster where a gene is located. The 1st number is predicted by antiSMASH, and the 2nd number is predicted by SMURF. Dashes indicate gene is not located in a predicted secondary metabolite cluster.

**Table 6:** RPKM expression values for genes upregulated in co-cultures compared to Tox 53 and Non-tox 17, and for genes highly upregulated in Non-tox 17 compared to Tox 53.

		30 h ^1^									72 h										
Gene ID ^2^	Chr ^3^	Tox 53			Non-Tox 17			Co-Culture			Tox 53			Non-Tox 17			Co-Culture			SM ^4^	Putative Function
(**a**) Genes which were upregulated in Co-cultures compared to Tox 53 and Non-tox 17																					
029700	2	3	±0.4	*	10	±2	**	8	±0.7	**	20	±2.2	*	27	±4	*	140	±6.8	**	0, 0	Peptidase
037820	2	75	±22	*	105	±37	**	78	±27	*	17	±2.2	*	51	±9.7	**	167	±3.6	***	0, 0	Hsp20 heat shock protein
124980	2	2	±0.1	*	3	±1.2	*	2	±0.3	*	6	±0.9	*	31	±12	**	88	±16	***	0, 0	Oxidoreduction decarboxylase
125000	2	0	±0	*	0	±0.1	*	0	±0.1	*	0	±0	*	6	±2.2	**	22	±4.6	***	0, 0	Membrane transport facilitator
126260	2	5	±0.5	**	1	±0.3	*	1	±0.5	*	1	±0.2	*	2	±0.3	**	9	±0.7	***	0, 0	FAD-dependent oxidoreductase
126390	2	10	±1	*	30	±3.2	***	18	±2.4	**	35	±0.5	*	74	±17	**	281	±24	***	0, 0	Metal-binding monoxygenase
135320	3	10	±1.3	*	50	±2.1	**	70	±3.3	***	55	±2.6	***	15	±0.8	*	33	±2.1	**	0, 0	Membrane transport facilitator
000840	4	1	±0.1	*	4	±0.4	**	7	±1.7	***	2	±0	*	27	±1.8	**	88	±35	***	0, 0	Membrane transport facilitator
000870	4	19	±3.2	*	57	±13	**	27	±3.1	*	2	±0.2	*	11	±1.1	**	26	±3.8	***	0, 0	Hypothetical protein
001010	4	0	±0	*	58	±2.5	***	56	±4.1	**	41	±2.5	**	28	±3.6	*	83	±2.5	***	0, 0	Cyt P450 oxygenase
013270	4	0	±0.1	*	1	±0.2	*	0	±0.1	*	1	±0.1	*	18	±8.9	**	80	±13	***	0, 0	4-oxalocrotonate tautomerase
013680	4	0	±0.1	*	1	±0.3	**	1	±0.1	**	1	±0.1	*	6	±0.4	**	12	±1.2	***	0, 0	Phospholipase C
016350	4	0	±0.1	*	0	±0.1	*	2	±1.6	**	0	±0.1	*	13	±1.6	**	23	±5.7	**	0, 0	NAD(P)H-dependent reductase
059120	5	0	±0	*	0	±0	*	0	±0	*	1	±0.1	*	1	±0.1	*	3	±0.5	**	0, 0	Metal binding fumarylacetoacetase
060770	5	0	±0.1	*	1	±0.1	**	1	±0.4	**	2	±0.1	*	26	±1.8	**	47	±3.1	***	0, 0	Acetate transport/EtOH synthesis
061090	5	0	±0.1	*	6	±0.4	***	4	±0.1	**	3	±0	*	2	±0.2	*	5	±0.5	**	0, 0	Serine/threonine MAP-kinase
091690	5	16	±0.9	*	24	±0.8	***	20	±0.5	**	23	±0.2	*	42	±3.7	**	94	±8.4	***	0, 0	Isocitrate lyase
096040	5	3	±0.2	*	73	±3.7	**	68	±8.6	**	11	±1.6	*	632	±110	**	1589	±530	***	0, 0 *	FAD-dependent oxidoreductase
096060	5	0	±0	*	1	±0.1	**	1	±0.1	**	2	±0.2	*	21	±5	**	95	±31	***	0, 0 *	Membrane transport facilitator
097430	6	17	±4.1	*	14	±0.4	*	11	±1.4	*	4	±0.6	*	11	±1.2	**	70	±32	***	0, 0	NAD-dependent dehydratase
097440	6	9	±0.8	*	10	±0.3	*	7	±0.9	*	3	±0.2	*	6	±1.3	**	32	±13	***	0, 0	Unknown-NAD(P) binding
040120	7	100	±13	*	302	±10	***	314	±23	**	352	±11	*	313	±22	*	665	±21	**	0, 0	FAD-binding oxidoreductase
117330	8	5	±0.3	*	94	±15	**	103	±5.6	***	24	±3.9	*	23	±3.4	*	71	±9.5	**	0, 0	Membrane transport facilitator
118940	8	0	±0	*	0	±0	*	0	±0	*	0	±0	*	3	±0.9	**	5	±2.7	***	8.5, 46	Polyketide synthase
118960	8	0	±0	*	0	±0	*	0	±0	*	0	±0	*	2	±0.6	**	5	±3.2	***	8.5, 46	Polyketide synthase
118970	8	0	±0.1	*	0	±0	*	0	±0	*	0	±0.1	*	4	±1.3	**	11	±5.6	***	8.5, 46	FAD-dependent oxygenase
118990	8	0	±0	*	0	±0	*	0	±0	*	0	±0	*	14	±4.3	**	46	±25	***	8.5, 0	Efflux pump, major facilitator
119000	8	0	±0	*	0	±0	*	0	±0	*	0	±0	*	65	±19	**	198	±120	***	8.5, 0	O-methyl transferase
122110	8	5	±0.2	*	2	±0.3	*	3	±0.6	*	3	±0.7	*	30	±5.1	**	136	±54	***	0, 0	Haem bifunctional peroxidase
(**b**) Genes which were highly overexpressed in Non-tox 17 compared to Tox 53																					
085640	1	0	±0	*	6	±0.3	**	5	±0.7	**	0	±0	*	5	±0.8	**	5	±1.2	**	0, 0	Peroxisome biogenesis
025220	2	0	±0	*	109	±7.5	**	81	±5.3	**	0	±0.4	*	103	±3.9	**	104	±7	**	0, 0	UPF0047 protein family
126420	2	1	±0.3	*	3	±0.2	**	5	±0.2	**	0	±0	*	5	±0.2	**	4	±0.2	**	0, 0	Protein glycosylation
060320	5	0	±0	*	5	±0.9	**	5	±0.9	**	0	±0	*	7	±0.6	**	6	±0.4	**	0, 0	Perforin domain
060350	5	0	±0	*	12	±1.6	**	8	±2	**	0	±0	*	2	±0.4	**	3	±0.5	**	0, 0	Unknown
062960	5	0	±0	*	7	±0.1	**	8	±0.1	**	0	±0	*	14	±0.8	**	13	±0.5	**	0, 20	Zn(2)-C6 transcription factor
062980	5	0	±0	*	12	±1.3	**	15	±0.5	**	0	±0	*	40	±2.2	**	36	±0.7	**	0, 20	Crotonase activity
062990	5	0	±0	*	5	±0.2	**	5	±0.1	**	0	±0	*	12	±0.3	**	10	±0.2	**	0, 20	Oxidase
095290	5	0	±0.3	*	90	±3.6	**	103	±4.3	**	1	±0.3	*	8	±0.5	**	13	±1.2	**	0, 0	Unknown
095300	5	5	±0.3	*	102	±9.4	**	97	±6.6	**	1	±0.2	*	6	±0.1	**	8	±1.1	**	0, 0	Protein-protein interactions
095800	5	4	±1.5	*	71	±3.1	**	80	±3.6	**	1	±0.2	*	62	±2.8	**	48	±0.4	**	0, 0	Short-chain reductase
066370	6	0	±0	*	12	±0.3	**	11	±0.8	**	0	±0	*	33	±1.9	**	34	±1.2	**	0, 0	Phosphorylation
008080	8	1	±0.3	*	216	±23	**	128	±14	**	65	±2.7	*	112	±4.6	**	128	±1.1	**	0, 0	Unknown
117340	8	0	±0	*	1	±0.2	**	3	±2.1	**	0	±0	*	4	±3.6	**	0	±0	**	0, 0	2-methylcitrate catabolism

^1^ Reads per kilobase per millions reads mapped (RPKM) were calculated for each 30 and 72 h Non-tox 17, Tox 53 and co-cultures reported as means and standard error. RPKM are measures of read abundance, which account for the total reads in a single replicate and individual gene length. This allows for better comparisons between treatments and genes rather than directly reporting reads. RPKM values for a single gene within 30 or 72 h with different numbers of asterisks *, ** or *** are statistically different based on α = 0.05. Values are color-scaled red for each gene and darker shades indicate more positive values scaled to the maximum within an individual gene; ^2^ Gene ID is the AFLA_# gene number (i.e., 029700 = AFLA_029700) designated in the *A. flavus* NRRL 3357 reference genome. Gene ID fonts colorcoded in orange, purple, blue, red or gray are in close proximity on chromosomes. The red gene numbers are in secondary metabolite clusters. Orange genes were not predicted by a secondary metabolite program but are involved in kojic acid synthesis; ^3^ Chr is the chromosome where each gene is located; ^4^ SM is the secondary metabolite cluster where a gene is located. The 1st number is the SM cluster predicted by antiSMASH, the 2nd number is the SM cluster predicted by SMURF.

**Table 7:** RPKM gene expression values for genes in imizoquin and aspirochlorine clusters.

		30 h ^1^									72 h									
Gene ID ^2^	Chr ^3^	Tox 53			Non-Tox 17			Co-Culture			Tox 53			Non-Tox 17			Co-Culture			SM ^4^
064230	6	30	±3	*	33	±0.9	*	21	±1.2	*	10	±1.6	*	209	±12.3	**	134	±4.7	**	0, 21
064240	6	6	±0.8	*	9	±0.7	*	7	±0.3	*	4	±0.3	*	68	±2	**	41	±1.4	**	0, 21
064250	6	31	±8.6	*	168	±3.9	*	79	±12.2	*	13	±0.9	*	613	±29.2	*	333	±6.2	*	0, 21
064260	6	4	±0.9	*	15	±1.3	*	9	±1.4	*	10	±0.5	*	153	±4.2	*	140	±1	*	0, 21
064270	6	37	±9.7	*	278	±9.2	*	141	±23.4	*	15	±1.1	*	1132	±36.9	*	711	±17.5	*	0, 21
064280	6	24	±7.3	*	150	±10.9	*	66	±10.2	*	6	±0.2	*	610	±24.1	*	290	±12.3	*	0, 21
064290	6	355	±90.6	**	615	±23.8	**	291	±42.5	*	43	±1.7	*	1393	±57.3	**	762	±31.9	**	0, 21
064300	6	15	±2.1	*	42	±0.8	*	20	±2.8	*	6	±0.4	*	146	±5.5	*	93	±1.2	*	0, 21
064310	6	5	±0.5	*	14	±0.1	*	8	±0.9	*	5	±0.3	*	59	±3.5	*	44	±1.2	*	0, 21
064320	6	18	±3.8	*	134	±7.8	*	60	±9.4	*	8	±0.6	*	443	±20.8	*	250	±4.7	*	0, 21
064330	6	7	±0.3	*	12	±1	*	7	±1.2	*	4	±0.3	*	23	±2.1	**	17	±1.5	**	0, 21
064340	6	6	±0.9	*	19	±0.3	*	9	±1.4	*	2	±0.3	*	22	±0.1	*	16	±1.5	*	0, 21
064350	6	0	±0.1	*	0	±0	*	0	±0	*	1	±0	*	1	±0.1	*	1	±0	*	0, 21
064360	6	0	±0.1	*	0	±0	*	0	±0.1	*	1	±0.1	*	1	±0	*	1	±0	*	0, 21
064370	6	1	±0.2	*	2	±0.1	*	1	±0.2	*	2	±0.1	*	4	±0.2	*	3	±0.2	*	0, 21
064380	6	185	±15.9	*	36	±2.3	*	63	±9.3	*	5	±0.3	*	85	±3.2	*	47	±1.8	*	0, 21
064390	6	11	±0.7	*	3	±0.4	*	5	±0.5	*	0	±0.1	*	7	±0.1	*	4	±0.2	*	0, 21
064400	6	32	±4.2	*	11	±0.5	*	18	±3.3	*	1	±0.2	*	21	±1.3	*	11	±0.4	*	0, 21
064410	6	9	±1.1	*	4	±0.7	*	5	±0.8	*	1	±0.1	*	8	±0.9	**	6	±0.4	**	0, 21
064420	6	112	±7.7	*	18	±0.4	*	22	±4.3	*	4	±0.6	*	46	±3.6	*	25	±1.5	*	0, 21
064430	6	89	±5.1	*	17	±1	*	22	±0.1	*	9	±0.8	*	38	±1.7	*	26	±1	*	0, 21
064440	6	35	±3	*	10	±0.7	*	16	±2.1	*	1	±0.1	*	22	±1.8	*	10	±0.8	*	0, 21
064450	6	44	±2.2	*	13	±1.3	*	21	±3.2	*	1	±0.2	*	33	±1.6	*	17	±0.8	*	0, 21
064460	6	16	±2	*	4	±0.6	*	8	±0.9	*	0	±0.1	*	9	±0.8	*	4	±0.4	*	1.1, 21
064470	6	32	±3.2	*	14	±0.9	*	20	±3	*	1	±0.2	*	20	±1.7	*	10	±0.1	*	1.1, 21
064480	6	137	±14.1	*	68	±7.1	*	81	±15.4	*	5	±0.2	*	79	±3.9	**	40	±2.9	**	1.1, 21
064490	6	90	±7.8	*	32	±1.2	*	46	±8.9	*	7	±0.4	*	60	±0.7	**	30	±1.4	**	1.1, 21
064500	6	727	±33.2	**	340	±28.1	*	309	±31.9	*	179	±5.1	*	996	±19.5	**	717	±16.8	**	1.1, 21
064510	6	40	±3.1	*	23	±0.8	*	34	±5.4	*	8	±0.5	*	32	±1.2	**	20	±0.1	**	1.1, 21
064520	6	40	±2.4	*	19	±1.6	*	29	±4.5	*	9	±0.4	*	33	±0.8	**	23	±0.5	**	1.1, 21
064530	6	64	±9.2	*	22	±2.7	*	29	±5.5	*	1	±0.1	*	34	±1.7	*	18	±0.8	*	1.1, 21
064540	6	24	±2.2	*	8	±0.9	*	11	±1.8	*	1	±0.1	*	17	±1.1	*	8	±0.4	*	1.1, 21
064550	6	22	±2.6	*	8	±0.9	*	14	±2.3	*	1	±0.1	*	20	±1.3	*	11	±0.3	*	1.1, 21
064560	6	6	±0.4	*	2	±0.1	*	5	±0.6	*	0	±0	*	10	±0.2	*	5	±0.1	*	1.1, 21
064570	6	56	±10.4	*	8	±0.5	*	12	±0.4	*	1	±0.2	*	16	±0.4	*	8	±0.2	*	1.1, 21
064580	6	122	±13.4	*	42	±4.6	*	53	±9.3	*	2	±0.4	*	67	±3.1	*	33	±2.1	*	1.1, 21
064590	6	122	±13.3	*	22	±1.5	*	32	±3	*	1	±0.1	*	42	±2.3	*	23	±0.6	*	1.1, 21
064600	6	7	±0.7	*	1	±0.3	*	2	±0.2	*	0	±0.1	*	4	±0.1	*	2	±0	*	1.1, 21
064610	6	2	±0.4	*	1	±0.2	*	1	±0.1	*	2	±0.1	*	7	±0.2	**	9	±2.8	**	1.1, 21

^1^ Reads per kilobase per millions reads mapped (RPKM) were calculated for each 30 and 72 h Non-tox 17, Tox 53 and co-culture, reported as means and standard error. RPKM are measures of read abundance, which account for the total reads in a single replicate and individual gene length. This allows for better comparisons between treatments and genes rather than directly reporting reads. RPKM values for a single gene within 30 or 72 h with different numbers of asterisks * or ** are statistically different based on α ≤ 0.05. Values are color-scaled red for each gene and a darker shade indicates more positive values that are scaled to the maximum within an individual gene. ^2^ Gene ID is the AFLA_# gene number (i.e., 029700 = AFLA_029700) designated in the *A. flavus* NRRL 3357 reference genome. Gene ID font color red and blue correspond to the imizoquin and aspirochlorine gene clusters, respectively. ^3^ Chr is the chromosome where each gene is located. ^4^ SM is the secondary metabolite cluster where a gene is located. The 1st number is the SM cluster predicted by antiSMASH, and the 2nd number is the SM cluster predicted by SMURF.

**Table 8:** Biological control mono and co-culture experimental design.

Cultured Isolates	Chemicals Extracted	Hours	Biological Replicates	Dishes per Rep
Non-tox 17	RNA and aflatoxin	30	5	9
Tox 53	RNA and aflatoxin	30	5	9
Co-culture of 17 + 53	RNA and aflatoxin	30	5	9
Non-tox 17	RNA and aflatoxin	72	4	1
Tox 53	RNA and aflatoxin	72	4	1
Co-culture of 17 + 53	RNA and aflatoxin	72	4	1
Non-tox 17	Aflatoxin	96	4	1
Tox 53	Aflatoxin	96	4	1
Co-culture of 17 + 53	Aflatoxin	96	4	1
Total samples			39	

*Aspergillus flavus* Non-tox 17 and Tox 53 isolates grew alone and together in co-cultures within separate Petri-dishes for 30, 72 and 96 h. Biological replicates at 30 h consisted of multiple Petri-dishes to accumulate adequate mycelial biomass for RNA extraction.

## Data Availability

Sequencing reads were submitted to NCBI’s Sequence Read Archive and can be accessed under BioProject ID PRJNA764255 and can be accessed at https://www.ncbi.nlm.nih.gov/sra. The remaining data is contained within the article and Supplementary Materials.

## Supplementary Materials

The following are available online at https://www.mdpi.com/article/10.3390/toxins13110794/s1, Table S1. Log2-fold changes for gene expression in Non-tox 17 versus (v) Tox 53, Co-culture vs. Tox 53, and Co-culture vs. Non-tox 17 at 30 and 72 h pair-wise comparisons if the fold change was ≥2 and *p*-values were ≤0.05; Table S2. Genes upregulated in Co-cultures compared to Tox 53 and Non-tox 17.
